# Fermented Dairy Food Intake and Risk of Colorectal Cancer: A Systematic Review and Meta-Analysis

**DOI:** 10.3389/fonc.2022.812679

**Published:** 2022-05-25

**Authors:** Zhi Liang, Xiaobiao Song, Jiang Hu, Riga Wu, Pengda Li, Zhenyu Dong, Lu Liang, Jijun Wang

**Affiliations:** ^1^ Department of General Surgery, Baotou Central Hospital, Baotou, Inner Mongolia, China; ^2^ Baotou Medical College, Baotou, Inner Mongolia, China

**Keywords:** fermented dairy foods, yogurt, cheese, probiotics, colorectal cancer, meta-analysis

## Abstract

It was highly controversial whether fermented dairy foods protect against colorectal cancer (CRC) because of conflicting results from current human epidemiologic studies; we therefore conducted this meta-analysis based on the case–control and cohort studies to estimate the holistic analyses. Finally, a total of seven case–control studies and ten cohort studies comprising a total of >20,000 cases were incorporated in the quantitative synthesis. Specifically, statistical evidence of significantly decreasing CRC risk in case–control studies was found to be associated with cheese intake (OR = 0.89, 95% CI = 0.82–0.97). In a subgroup analysis, cheese intake was correlated with lower colon cancer (OR = 0.89, 95% CI = 0.79–1.00) and rectal cancer (OR = 0.86, 95% CI = 0.74–1.00) risk in case–control studies. Furthermore, we also found that the higher intake of yogurt may lower the risk of rectal cancer (OR = 0.75, 95% CI = 0.65–0.88) in cohort studies. The consumption of fermented dairy foods may be relevant to decrease CRC risk in this meta-analysis.

**Systematic Review Registration:**

https://www.crd.york.ac.uk/prospero/display_record.php?ID=CRD42021269798, CRD42021269798.

## Introduction

In 2020, colorectal cancer (CRC) was the third most leading cause of cancer death in the Western world. Results of epidemiological studies show that a multitude of risk factors are relevant to colorectal cancer, including a lifestyle, diet, genetics, and obesity, and diet played a pivotal role for the disease (1). There was a general consensus in the diet with colorectal cancer—high red meat and processed meat consumption has been consistently associated with an increased risk of developing colorectal cancer; however, dietary fiber intake can protect from colorectal cancer (2). In addition, with the explosion of food processing in technologies, better seeking of new risk factors in diet associated with colorectal cancer is necessary to prevent the disease. Currently, a wide variety of milk and dairy products are consumed by over 6 billion people worldwide (3); against this background, greatly meeting the increasing demand for wide-ranging practical value in novel dairy products had attracted a great deal of attention in clinical practice. Fermented dairy foods were a traditional fresh dairy fermented by complex microorganisms, generating a significant amount of probiotics (4). The latter played an essential role in modulating the host gut microbiota for preventing carcinogenesis (5). Yogurt and cheese were exemplified in fermented dairy foods.

Increasing data had supported a role for the imbalances gut microbiota played on colorectal carcinogenesis (6). Multiple studies have lately shown that the gut microbiota dysbiosis can be remodeled through short-term effects of probiotic-enriched dietary intervention (7). Studies have also found recently that the consumption of fermented dairy foods was closely linked to colorectal cancer, yet the overwhelming majority of analyses were the consequences of negative or neutral results from the previous systematic review and meta-analysis (8–10). These apparent differences between theoretical and experimental results were intriguing but require validation. Since dietary interventions were the most practical and economical approach than other treatment modalities, further analysis was demanded.

In this study, yogurt and cheese were chosen typically represented on fermented dairy foods in order to ascertain the concrete links in colorectal cancer. We developed this comprehensive meta-analysis on published cohort and case–control studies according to the PROSPERO guidelines https://www.crd.york.ac.uk/prospero/display_record.php?ID=CRD42021269798 to evaluate the impact of the fermented dairy foods intake on colorectal cancer (CRC).

## Methods

### Publication Search and Inclusion Criteria

Three databases (PubMed, Embase, Web of Science) were searched for all articles in English language since database inception in July 2021. We employed the following terms in the analysis: “fermented food or fermented milk or cultured milk or cheese or yogurt or lactic acid bacteria” and “Colorectal Neoplasm” or “Colorectal Cancer” or “Colonic Cancer” or “Rectum Cancers” and so on. The study design was not restricted during the retrieval of process in order to gain a comprehensive search of literature.

If a study meets the following criteria, the results could be incorporated for inclusion in the meta-analysis: (1) a topic of the association about yogurt or cheese consumption and CRC, colon, or rectal cancer risk; (2) the outcome relied on dietary information from questionnaires; (3) odds ratio (OR), hazard ratio (HR), and relative risk (RR) with 95% CIs can be acquired through reading full-text articles; (4) original articles were published in English; and (5) the study of design was a cohort or case–control study. In addition, we may exclude some articles that meet our exclusion criteria. (1) Duplicate articles in different databases; (2) cell or animal experiments; (3) meta-analysis studies; (4) reviews, letters, and commentaries; and (5) articles lacking specific data.

### Data Extraction

First of all, duplicate literature from the databases was removed from this study. Then, two authors (ZL and JW) independently screened the titles and abstracts to exclude some works of data that did not meet our eligibility. In parallel, based on the inclusion and exclusion criteria, the outcome of specific information can be acquired through reading the full texts, such as first author of the works, year of publication, sex, country of recruitment, follow-up period, dairy type, and the number of cases or controls.

### Study Quality Assessment

The Newcastle–Ottawa Scale (NOS) was performed for some cohort or case–control studies. Two reviewers (ZL and JW) determined the quality of the included studies independently. The Newcastle–Ottawa Scale (NOS) of the maximum score was 9, and a high score (≥6) indicated high quality in this study. If there were any discrepancies, disagreements were addressed through discussion.

### Statistical Analysis

We calculated the consumption of yogurt or cheese in the highest compared with the lowest categories to computer odds ratios or (case–control studies) rr and hr (cohort studies) corresponding to the 95% confidence interval (95% CI). The study of heterogeneity was assessed with the Cochran’s Q statistic and I^2^ statistics. If I^2^ ≥ 50% from the statistics analysis, the fixed-effects model was performed for calculation; otherwise, the random-effects model was employed. In order to explore the sources of heterogeneity, we also performed a sensitivity analysis by logistic meta-regression analyses. In addition, we examined prespecified stratified analyses for different study characteristics: region, dairy type, sex, and tumor location (colorectal cancer, colon cancer, proximal or distal colon cancer, and rectal cancer). The funnel plot and Begg’s rank correlation method were employed to assess publication bias.

The statistical analyses were performed by STATA 16.0 software (Stata Corp, College Station, TX).

## Results

### Study Selection

**Figure 1:**
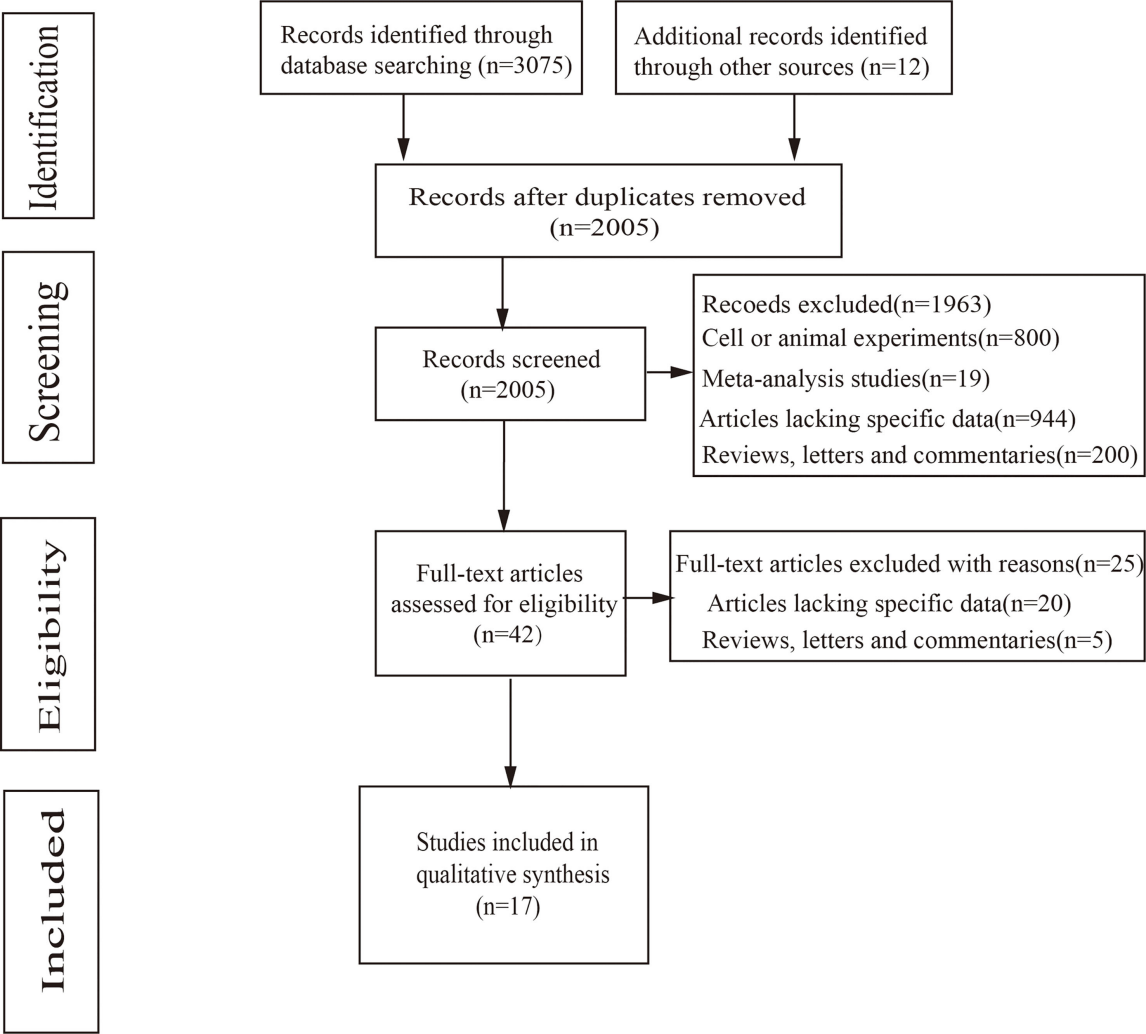
Flowchart for identification of studies.

The flow diagram of the steps was presented as a flowchart in **Figure 1**. Of the 17 reports remaining after 2005 abstracts were screened, the studies were included in the meta-analysis: 10 prospective cohort studies (11–20) and 7 case–control studies (21–27).

### Study Characteristics

The main characteristics of the studies are depicted in **Table 1** and **Table 2**. Simultaneously, 6,968 cases and 8,536 controls were included in the case–control studies (**Table 1**). A total of 1,310,276 participants with 14,944 cases were recorded in this cohort studies (**Table 2**). The case–control studies were conducted in five countries (Netherland, Moroccan, France, the United States, Canada). In addition, for the cohort studies, many countries were incorporated in this analysis, including 2 from the United States, 3 from Sweden, 1 from Italy, 1 from Japan, and 2 from each of the 10 different European countries. Two types of studies were conducted in adults.

**Table 1:** Characteristics of literatures included in the meta-analysis.

Reference	Study characteristics (age, y)	No. of cases and endpoint	Sex,no. of cases(M/W)	No. controls and type	Exposure	OR	Adjustments to OR	Funding source	Outcome	NOS quality score
Kampman et al. 1994 (21)	Netherlands (up to 75 at age of diagnosis)	232CC	NA	520H	Fermented dairy products > 242VS< 22g/d;Yogurt:> 91g/D vs Non-users; Hard cheese:> 49VSc 19g/d	0.86(0.51,1.44);1.16(0.71,1.88);1.21(0.72,2.03)	Adjusted for age,gender, urbanization level,family history, cholecystectomy, total energy intake, energy-adjusted intake of fat, dietary fibre, vitamin C and alcohol	Agency	Incidence	7
Kinany et al. 2020(22)	Moroccan ( more than 18 years old)	1453CRC	(49.3%/50.7 %)	1453H	Yogurt >44.00 VS<44.00 g/day Cheese >12.00 g/day VS<12.00 g/day	CRC 0.74 (0.64-0.86) CC 0.72 (0.58-0.89) ;R 0.76 (0.61-0.93)/CRC 0.89 (0.79-1.00);CC 0.91 (0.77-1.06);R 0.88 (0.75-1.04)	Multivariable model: conditional logistic regression using age in years, residence (urban, rural), education level (illiterate, primary, sec ondary, higher), monthly income (low, medium, high), physical activity intensity (high, moderate and low), smoking status (never smoker, Ex smoker and current smoker), BMI categories (normal, underweight, overweight, obesity), non-steroidal anti-infammatory drugs (yes or no),	Agency	Incidence	7
Boutron et al. (24)	France (30-75)	171CRC	(109/62)	309H	Cheese Q5VSQ1;	CRC 1.2(0.6-2.2)	total energy intake (continuous/Kcal), intakes of red processed meat and dietary fber (both continuous-g/day), family history of colorectal can cer (yes logarithmic or no) transformation for equality of variance and multiple logistic regression controlling for age, sex and caloric intake.	Agency	Incidence	8
Shannon et al. (23)	US (30-62)	424CRC	(238/186)	414H	Yougrt:>1 VS 0 servings/week	CRC(M) 1.27 (0.69-2.36) ;CRC(W)0.65 (0.37-1.16)	adjusted for age and total energy	Agency	Incidence	8
Kampman et al. (25)	US (30-70)	1983 CC	(1095/888)	2400H	Cheese:High VS Low intake; Yogurt:High VS Low	CC(M) 0.9 (0.7-1.2) ;(W) 0.8 (0.7-1.1)/CC (M) 1.0 (0.8-1.2); (W) 1.1 (0.9-1.3)	Adjusted for age, BMI, family history of first-degree relative with colorectal cancer, use of aspirin, use of NSAIDs, energy intake, long-term vigorous physical activity and dietary fiber	Agency	Incidence	7
Williams et al. (26)	US (40-79)	945 R (Whites +African-Americans)	NA	959 H	Cheese (Whites):Q4VSQ1;Yogurt(Whites):Q2VSQ1;Cheese(African-Americans):Q4VSQ1; Yogurt(African-Americans):Q4VSQ1 (servings/wk)	0.73(Whites)(0.50-1.06);0.69(Whites)(0.53-0.89);1.04(African-Ameri cans)(0.44-2.46); 1.08(African-Americans)(0.62-l .87)	Adjusted for age, sex, education, income, BMI 1 year ago, physical activity, family history, nonsteroidal anti-inflammatory drug use, and total energy intake.	Agency	Incidence	7
Zhuoyu et al. (27)	11 Canada (20-74)	1760 CRC(ON+NL)	NA	2481 H	Cheese (NL):Q5VSQ1;Cheese(ON):Q5VSQ1;Yogurt(NL):Q3VSQ1;Yogurt(ON):Q5 VS Q1	1.25(NL)(0.76,2.05); 0.90(ON)(0.70,1.14)/ 1.02(NL)(0.75,1.39); 0.85(ON)(0.68,1.07)	Adjusted for total energy intake, age, sex, BMI, physical activity (METs/week), first-degree relatives with CRC, polyps, diabetes, reported colon screening procedure, cigarette smoking, alcohol drinking, education attainment, household income, marital status, regular use of NSAID, regular use of multivitamin supplements, reported HRT (females only), and intakes of fruits, vegetables and red meat. Variables were included in the final model based on a >10% alternation in the parameter coefficient of interest.	Agency	Incidence	7

CRC, colorectal cancer; CC, colon cancer; DC, distal colon cancer; R, rectal cancer; M, man; W, woman; NL, subjects in Newfoundland and Labrador; ON, subjects in Ontario.

**Table 2:** Characteristics of literatures included in the meta-analysis.

Reference	Study cohort and characteristics (age, y)	No. of participants (M/W)	No. of incident cases	Outcome (Incidence/ Mortality)	Follow-up length, y	Exposure	RR/HR	Adjustments to the RR/HR	Funding source	NOS quality score
Kearney et al. (11)	USA: Health Professionals Follow-up Study (40-75)	47,935 M	203 CC	Incidence.	6	Hard cheese: >1/d vs <1/mo (1 slice)	RR CC: 1.35 (95% CI: 0.67, 2.75)	Age, total calories, family history of colon cancer, previous polyp, screening, past history of smoking, alcohol consumption, aspirin use, physical activity, BMI, red meat, saturated fat, and dietary fiber intakes	Agency industry	8
Singh et al. (12)	USA: Adventist Health Study (25-100)	32051	157 CC	Incidence.	6	Cheese (excluding cottage cheese): **>**2 servings/wk vs never	RR CC: 1.04 (95% CI: 0.69, 1.59)	Age at baseline, sex, BMI, physical activity, parental history of colon cancer, current smoking, pasts smoking, alcohol consumption, and aspirin use	Agency	7
Terry et al. (13)	Sweden: Swedish Mammography Screening Cohort (median 55)	61,643 W	572 CRC, 371 CC and 191 R	Incidence.	11.3	Fermented dairy servings/mo (yogurt and cultured milk): Q4 vs Q1	RR CRC: 0.90 (95% CI: 0.72, 1.13) RR CC: 0.76 (95% CI: 0.57, 1.01) RR R: 1.28 (95% CI: 0.87, 1.89)	Age, BMI, education level, total energy and quartiles of red meat, alcohol, and energy-adjusted folic acid and vitamin C intake. Individual dairy products were mutually adjusted	Agency	7
Larsson et al. (14)	Sweden: Swedish Mammography Cohort (40-76)	60,708 W	798 CRC, 543 CC(246 PC, 170 DC,127 unknown), 249 R	Incidence.	14.8	Cheese: **>**3 vs <1 serving/d	RR CRC: 0.65 (95% CI: 0.44, 0.96) RR PC: 0.76 (95% CI: 0.39, 1.50) RR DC: 0.24 (95% CI: 0.07, 0.82) RR R: 0.89 (95% CI: 0.46, 1.71)	Stratified by age at recruitment and the year of entry into the cohort. Adjusted for age, BMI, education, total energy intake and quintiles of intakes of folate, vitamin B-6, cereal fiber and red meat	Agency	7
Larsson et al. (15)	Sweden: Cohort of Swedish Men (45-79)	45,306 M	449CRC, 276 CC and 173 R	Incidence.	6.7	Cultured milk (sour milk and yogurt): **>**1 serving/d vs never/Hard cheese: **>**3 slices/d vs	RR CRC: 1.07 (95% CI: 0.86, 1.34); RR CC: 1.17 (95% CI: 0.88, 1.56) RR R: 0.94 (95% CI: 0.66, 1.33)/RR CRC: 0.79 (95% CI: 0.56,	Stratified by age at baseline. Adjusted for education, family history of CRC, BMI, exercise, history of diabetes, cigarette smoking, aspirin use, multivitamin supplement use, total energy and quartiles of saturated fat, total vitamin D, alcohol, fruit, vegetable, and red meat intake	Agency	7
Valeria et al., 2011 (16)	Italy: Italian European Prospective Investigation into Cancer and Nutrition cohort (EPIC-Italy cohort) (mean of 51)	14,178/31,063	289 CRC (215 CC and 74 R)	Incidence.	12	<4 Yogurt: T3 vs T1 slices/wk (median intake)	RR CRC (entire cohort): 0.65 (95% CI: 0.48, 0.89) RR CRC (M): 0.47 (95% CI: 0.28, 0.81) RR CRC (W): 0.69 (95% CI: 0.47, 1.03)	Stratified by diet questionnaire. Adjusted for energy, animal fat, red meat intake, dietary calcium, dietary fiber, simple sugars, BMI, alcohol consumption, smoking, education level,recreational activity (excluding sports),sporting and type of work.	Agency	7
Neil et al., 2013 (17)	10 European countries (Denmark, France, Germany, Greece, Italy, the Netherlands, Norway, Spain, Sweden, and the	142,141/334, 981	4513 CRC, 2868 CC and 1645 R	Incidence.	11	Yogurt (natural and flavored yogurt in all cohorts, and, additionally, fermented milk in Sweden, Norway, and Denmark)	RR CRC: 0.90 (95% CI: 0.81, 0.99) RR CC: 0.88 (95% CI: 0.77, 1.00) RR R: 0.93 (95% CI: 0.79, 1.10)	Stratified by age (1-y categories), sex and center. Adjusted for total energy intake, BMI, physical activity index, smoking status and intensity, education status, ever-use of contraceptive pill, ever-use of HRT, menopausal status, alcohol consumption, intakes of red and processed meat and fiber.	Agency	7
	United Kingdom): European Prospective Investigation into Cancer and Nutrition (EPIC)									
Laura et al., 2018 (18)	Spain: PREDIMED trial (55-80)	7216	97 CRC	Incidence.	6	Cheese (includes all types of cheese: petit suisse, ricotta,cottage, spreadable, and semicured/cured cheeses): 44 vs 11 g/d	RR CRC: 1.23 (95% CI: 0.74, 2.06)	Stratified by recruitment center. Adjusted for intervention group, sex, age, leisure time physical activity, smoking status, family history of cancer, education level, history of diabetes, use of aspirin treatment andcumulative average consumption of vegetables, fruits, legumes, cereals, fish, meat, olive oil and nuts, and alcohol.	Agency	7
Matsumoto et al. (19)	Japan: Jichi Medical School (JMS) Cohort Study(18-90)	11606	25CC	Mortality	9.15	yogurt	HR Colon 1.28 ( 95% CI:0.30 - 5.48 )	adjusted by sex and age	Agency	7
Dik et al. (20)	10 European countries(Denmark,France, Germany, Greece, Italy, the Netherlands, Norway, Spain, Sweden, and United	521448	1525CRC	Mortality	8	yoghurt Q4vsQ1 ;cheese Q4vsQ1	HR CRC 1.09 95% CI, 0.88–1.34 HR CRC 0.93 95% CI, 0.76–1.14	adjusted for age at colorectal cancer diagnosis (continuously per one year increase), sex, prediagnostic ,BMI (continuous), smoking status (never, former, current, unknown), and energy intake (continuous).	Agency	8
	Kingdom):European Investigation into Cancer and Nutrition (EPIC) cohort.(25-70)									

CRC, colorectal cancer; CC, colon cancer; DC, distal colon cancer; R, rectal cancer; M, man; W, woman; NL, subjects in Newfoundland and Labrador; ON, subjects in Ontario.

### Quantitative Synthesis

**Figure 2:**
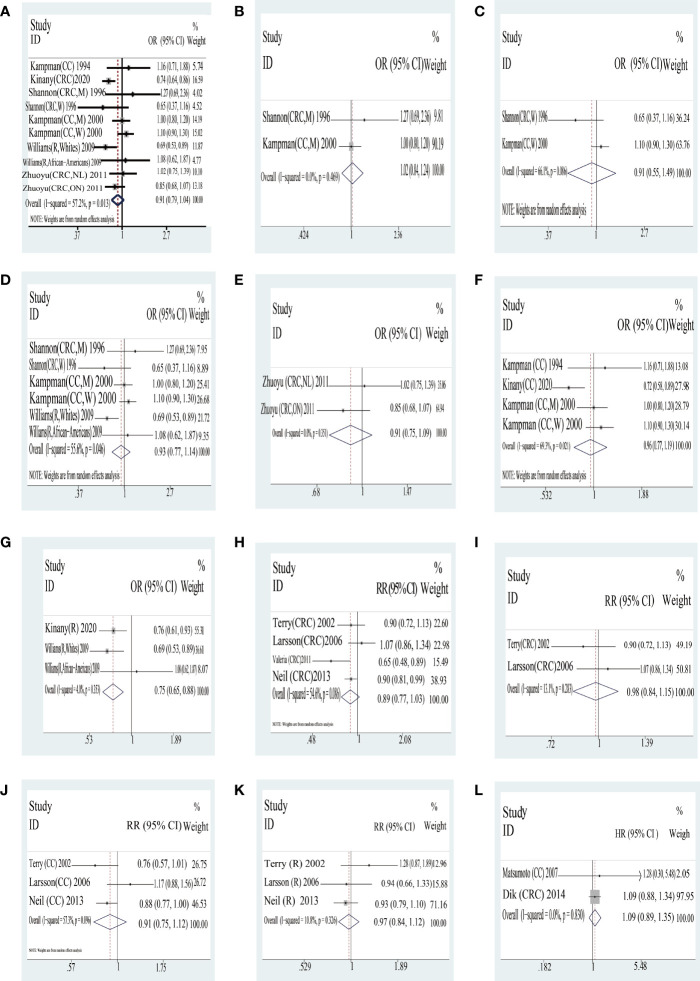
Yogurt: forest plot of case–control studies **(A–G)** and cohort studies **(H–K)** in yogurt examining the association between consumption of yogurt and risk of colorectal cancer as well as the consumption of yogurt in the mortality of colorectal cancer **(L)**. CRC, colorectal cancer; CC, colon cancer; DC, distal colon cancer; R, rectal cancer; M, man; W, woman; NL, subjects in Newfoundland and Labrador; ON, subjects in Ontario. Case–control studies: **(A)** total CRC; **(B)** CRC in man; **(C)** CRC in woman; **(D)** US; **(E)** Canada; **(F)** colon cancer; **(G)** rectal cancer; Cohort studies: **(H)** total CRC; **(I)** Sweden; **(J)** colon cancer; **(K)** rectal cancer; **(L)** mortality of CRC.

*Yogurt* The outcome of the high consumption compared with low consumption on yogurt is shown in **Table 3** and **Figure 2**. In case–control studies, we included seven quantitative studies to assess the joint association of yogurt consumption with colorectal cancer which was not statistically significant in the outcome (OR = 0.91, 95% CI = 0.79–1.04), as is shown in **Table 3**, in terms of subgroup analysis by region, sex, and tumor location. The results from subgroups of region and sex had no statistical difference; however, there were two different outcomes on tumor location, rectal cancer (OR = 0.75, 95% CI = 0.65–0.88) and colon cancer (OR = 0.96. 95% CI = 0.77,1.19). In cohort studies, we enrolled 10 quantitative studies to analyze the incidence and mortality between consumption of yogurt and colorectal cancer. In addition, we also formed subgroups regarding region and tumor location, as shown in **Table 3**. Overall, yogurt intake levels were not statistically significant in mortality (HR = 1.09, 95% CI = 0.89,1.35) and incidence (RR = 0.89, 95% CI = 0.77,1.03).

**Table 3:** Yogurt.

SubgroupStudies, n		heterogeneity		OR/RR/HR (95% CI)	P-value
		I^2^ (%)	P-value		
Case–control studies				OR (95% CI)	
Total yogurt	10	57.2%	0.013	0.91 (0.79,1.04)	>0.05
Canada	2	0.0%	0.351	0.91 (0.75,1.09)	>0.05
US	6	55.6%	0.046	0.93 (0.77,1.14)	>0.05
Men	2	0.0%	0.469	1.02 (0.84,1.24)	>0.05
Women	2	66.1%	0.086	0.91 (0.55,1.49)	>0.05
Colon cancer	4	69.3%	0.021	0.96 (0.77,1.19)	>0.05
Rectal cancer	3	4.0%	0.353	0.75 (0.65,0.88)	<0.05
Cohort studies				RR (95% CI)	
Total yogurt	4	54.6%	0.086	0.89 (0.77,1.03)	>0.05
Sweden	2	13.1%	0.283	0.98 (0.84,1.15)	>0.05
Colon cancer	3	57.3%	0.096	0.91 (0.75,1.12)	>0.05
Rectal cancer	3	10.8%	0.326	0.97 (0.84,1.12)	>0.05
				HR (95% CI)	
Total yogurt	2	0.0%	0.830	1.09 (0.89,1.35)	>0.05

**Figure 3:**
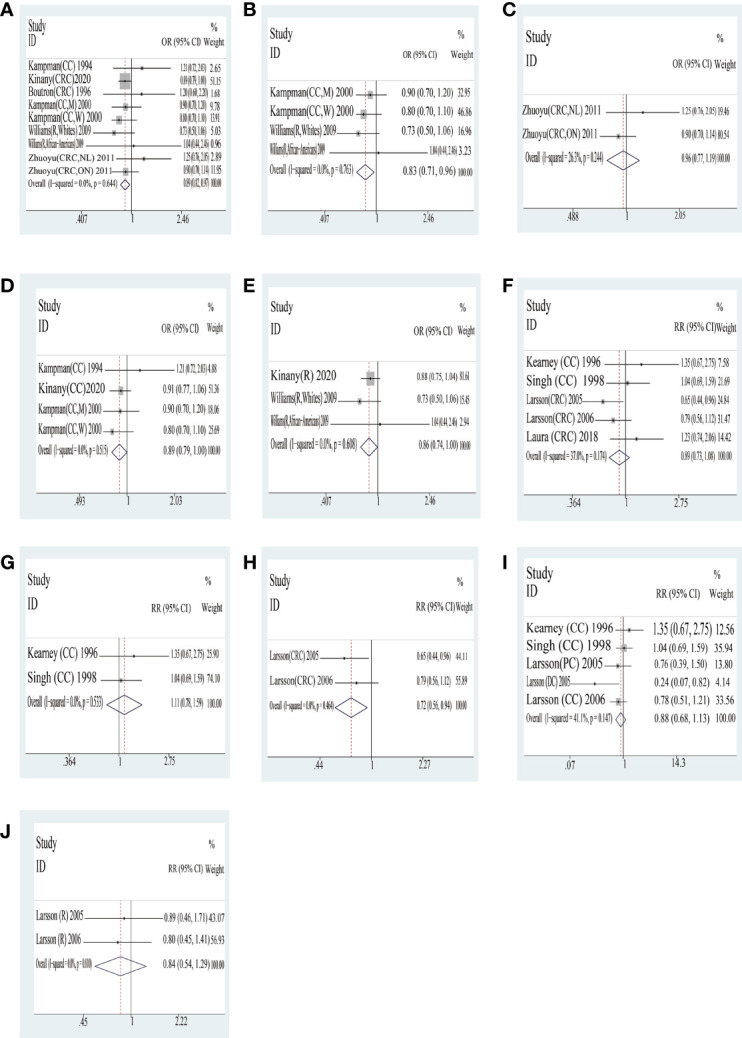
Cheese: forest plot of case–control studies **(A–E)** and cohort studies **(F–J)** in yogurt examining the association between consumption of yogurt and risk of colorectal cancer. CRC, colorectal cancer; CC, colon cancer; DC, distal colon cancer; R, rectal cancer; M, man; W, woman; NL, subjects in Newfoundland and Labrador; ON, subjects in Ontario. Case–control studies: **(A)** total CRC; **(B)** US; **(C)** Canada; **(D)** colon cancer; **(E)** rectal cancer. Cohort studies: **(F)** total CRC; **(G)** US; **(H)** Sweden; **(I)** colon cancer; **(J)** rectal cancer.

*Cheese* The outcome of the high consumption compared with low consumption on yogurt is shown in **Table 4** and **Figure 3**. In case–control studies, seven quantitative studies were included to analyze the joint association of cheese consumption with colorectal cancer. The result of the consumption of total cheese with colorectal cancer was a statistical difference (OR = 0.89, 95% CI = 0.82,0.97). In addition, there was a statistically significant difference in tumor location with cheese of consumption between colon cancer (OR = 0.89, 95% CI = 0.79,1.00) and rectal cancer (OR = 0.86, 95% CI = 0.74,1.00). However, systematic analyses of different countries based on extant data were not statistically significantly different. In cohort studies, interestingly, no statistically significant differences in outcome were found; only one country (Sweden) was a statistically significantly different in consumption of yogurt (RR = 0.72, 95% CI = 0.56,0.94).

**Table 4:** Cheese.

SubgroupStudies, n		heterogeneity		OR/RR/HR (95% CI)	P-value
		I^2^ (%)	P-value		
Case–control studies				OR (95% CI)	
Total cheese	9	0.0%	0.644	0.89 (0.82,0.97)	<0.05
US	4	0.0%	0.763	0.83 (0.71,0.96)	>0.05
Canada	2	26.3%	0.244	0.96 (0.77,1.19)	>0.05
Colon cancer	4	0.0%	0.515	0.89 (0.79,1.00)	<0.05
Rectal cancer	3	0.0%	0.608	0.86 (0.74,1.00)	<0.05
Cohort studies				RR (95% CI)	
Total cheese	5	37.0%	0.174	0.89 (0.73,1.08)	>0.05
US	2	0.0%	0.533	1.11 (0.78,1.59)	>0.05
Sweden	2	0.0%	0.464	0.72 (0.56, 0.94)	<0.05
Colon cancer	5	41.1%	0.147	0.88 (0.68, 1.13)	>0.05
Rectal cancer	2	0.0%	0.810	0.84 (0.54, 1.29)	>0.05

### Evaluation of Heterogeneity

We will consider heterogeneity among studies in overall comparisons and choose the random-effects model (P heterogeneity< 0.001 and I^2^ > 50%.). In order to comprehensively analyze the outcome, we formed subgroups on sex, countries, and tumor location.

### Sensitivity Analysis

In order to understand the meta-stability of the associations observed, we omit one study at a time from the outcome in cohort and case–control studies. If the observation did not appreciably change, we confirmed the reliability of the data analysis.

### Publication Bias

**Figure 4:**
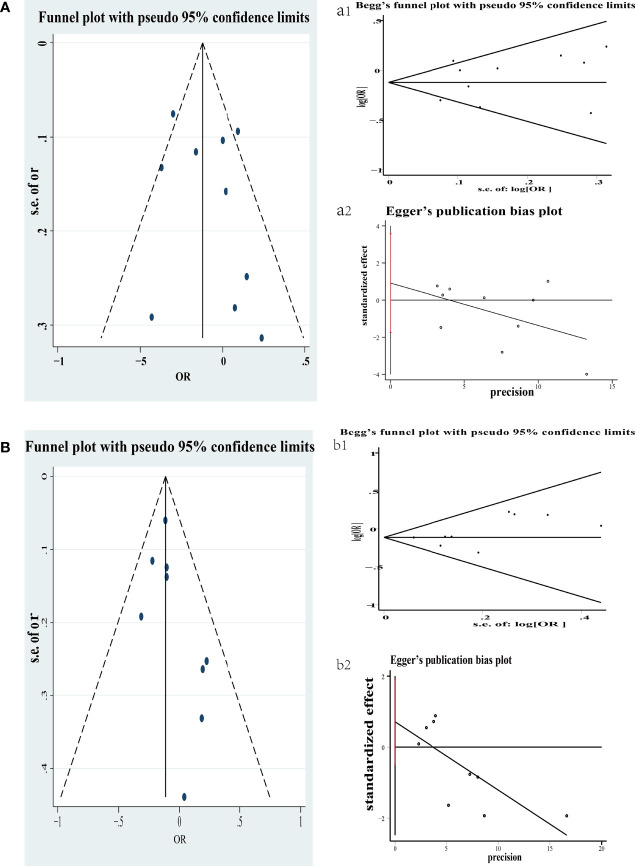
Funnel plot of colorectal cancer risk associated with consumption of yogurt in case–control **(A)**; Begg’s and Egger’s funnel plot for publication bias test on consumption of yogurt in case–control (a1 and a2). Each point represents a separate study for the indicated association. s.e., standardized effect. Funnel plot of colorectal cancer risk associated with consumption of cheese in case–control **(B)**; Begg’s and Egger’s funnel plot for publication bias test on consumption of cheese in case–control (b1 and b2). Each point represents a separate study for the indicated association. s.e., standardized effect.

The publication bias of selecting literature was evaluated by Begg’s test. **Figure 4** exhibits two funnel plots of yogurt and cheese of case–control included in the meta-analysis. There was no apparent publication bias in yogurt (P = 1.000) and cheese (P = 0.251).

## Discussion

Here, we developed this meta-analysis on 17 cohorts and case–control studies with more than 1,310,276 participants and 14,944 cases. By comparing the high consumption with low consumption in two distinct study designs, we attempted to identify the link of fermented food such as yogurt and cheese in association with colorectal cancer. We discovered new associations, some of which have not previously been published. In general, whereas our results from this meta-analysis are consistent with the observation that the consumption of yogurt and cheese remained unclear connection for previous studies, we discovered some novel links in different study designs and subgroups.

For example, we observed that different primary tumor sites were associated with yogurt consumption. Results of the case–control study found yogurt intake to be associated with a decreased risk of rectal cancer, but not colon cancer. The reason for this disparity in the outcome was unclear. Furthermore, we found that there is an inverse association between dietary intake of cheese in case–control and the risk of colorectal cancer. There are several reasons that could account for these outcomes. Compared to other fermentation products such as yogurt, having greater viable probiotics in cheese is advantageous. The reason for these differences was interpreted with unique characteristics in higher pH and buffering capacity and lower oxygen and salt levels. In these settings, the long-term survival of probiotics was observed in the center of the cheese. Theoretically, it may also play a protective role in storage and passage through the gastrointestinal tracts (28). From the field of microbial ecology perspective, it is suggested that the administration of sufficient amounts of diet rich in probiotics may be associated with a lower incidence of colorectal cancer (29). There was a marked regional difference in consumption of cheese such as Sweden from Europe which was distinct from other countries. Simultaneously, consumption of cheese in a cohort study is negatively associated with the risk of colorectal cancer. Swedish people are well known to have healthy and fixed dietary habits, particularly breakfast, at which they prefer to consume them as daily dietary activities, so they gain more probiotics from cheese compared to other countries. This resulted in a lower incidence of CRC in Swedish people.

**Figure 5:**
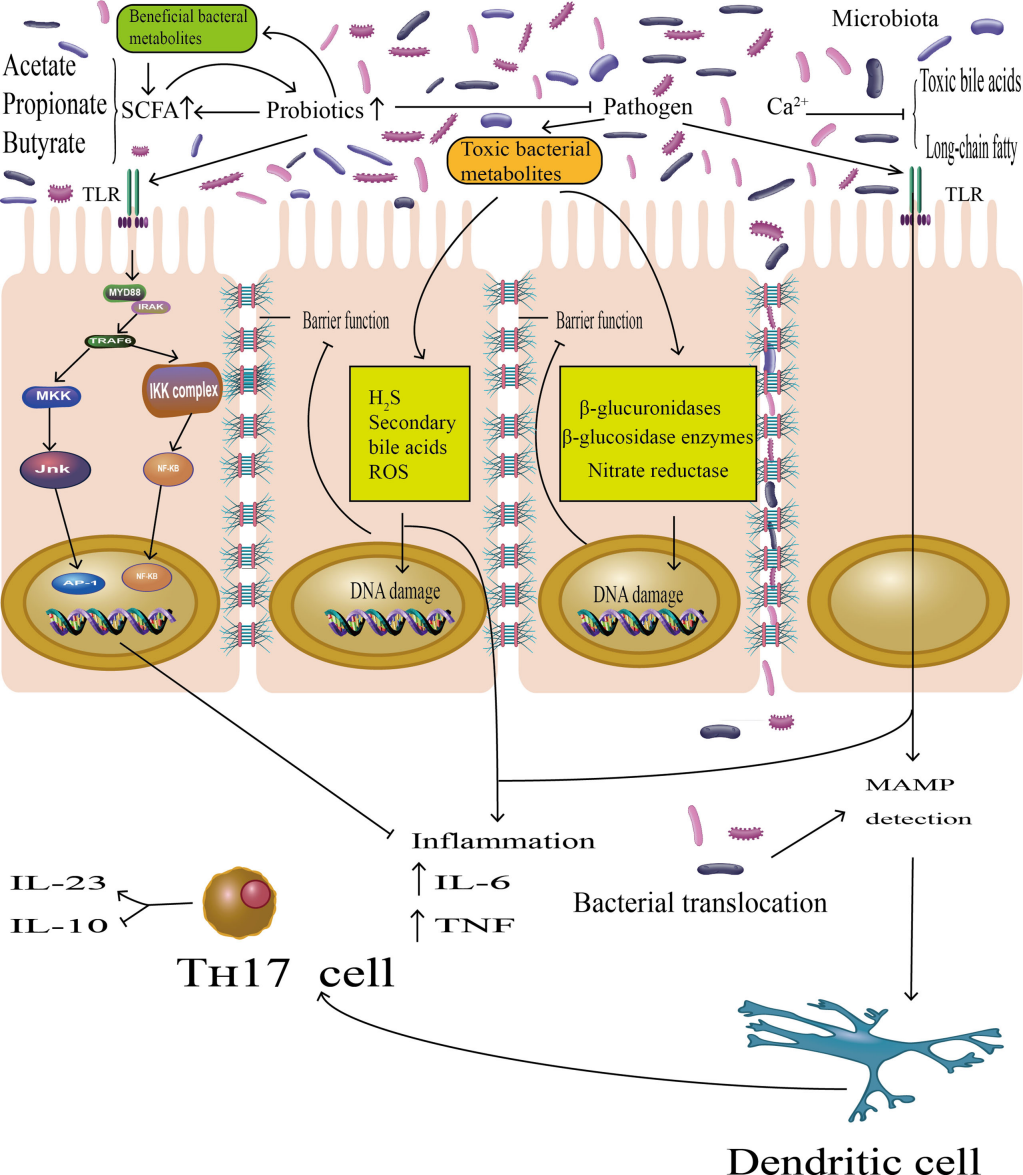
The probiotics play essential roles in host metabolism, immune modulation, and colonization resistance to pathogens, suppressing the CRC progression. On the one hand, there were studies demonstrating that probiotics can prevent the attachment of pathogenic bacteria to gut epithelia. On the other hand, short-chain fatty acids (SCFAs), mainly acetate, butyrate, and propionate, are major metabolic products of probiotics, promoting probiotics growth and reproduction, protecting the intestinal barrier function. Probiotics may represses toxic bacterial metabolites by indirectly inhibiting the growth of pathogens. Toxic bacterial metabolites can induce DNA damage in epithelial cells; indirectly impaired barrier function was among the constellation of accepted pathologies in CRC and generated local or chronic inflammation by producing inflammatory cytokines (IL-6, TNF). In addition, pathogenic bacteria may also exert pro-inflammatory effects *via* microorganism-associated molecular patterns (MAMPs) by Toll-like receptors (TLRs), which lead to detection by dendritic cells (DC) as well as activation of Th-17 cells, and the latter will promote the expression of the pro-inflammatory mediator IL-23 and block the expression of the anti-inflammatory mediator IL-10. However, probiotics also bound the Toll-like receptor (TLR), which activated the TLR–NF-kB signal transduction pathway to inhibit the inflammatory effects.

Epidemiologic studies have shown that fermented dairy foods such as yogurt and cheese, the main sources of probiotics in human diets, have proved to be one source of calcium in Western populations (30). Therefore, there are several possible reasons that could explain the consumption of fermented dairy foods associated with CRC. A recent meta-analysis showed that dietary patterns rich in calcium in dairy foods may decrease the incidence of colorectal adenomas, which was precancerous lesions of colorectal cancer (31). The underlying mechanism could arrest excessive proliferation and mutation in the gut epithelium by binding to toxic bile acids and long-chain fatty acids (32). Some studies argued that high physiological concentrations of bile acids in the colorectal epithelium may initiate carcinogenesis (**Figure 5**) (33). Moreover, probiotics in fermented dairy foods may also play a pivotal role in colorectal cancer. There have been some animal models of evidence which have suggested that probiotics can competitively adhere to intestinal mucus to prevent colonization of pathogens (34). Probiotics may regulate the imbalance of intestinal microflora to suppress tumorigenesis *via* multiple mechanisms (**Figure 5**) (35–40). Hence, consumption of calcium in fermented dairy foods may decrease the incidence of CRC and lower the risk of developing colorectal tumors.

In this meta-analysis, there were some inadequacies, although we enrolled a great deal of high-quality studies. No apparent publication bias has not been perceived, yet its impact may remain. Besides this, there was a marked heterogeneity throughout this analysis. The reason could be attributed to the difference in food products, regular and prolonged dietary habits, and the sample size of this study. Finally, the smaller sample sizes of the relevant analysis allows us not to fully account for mortality in colorectal cancer. We hope more experimental and theoretical evidence will be able to verify the outcome.

In conclusion, our meta-analysis suggests that fermented dairy food intake may have an impact on the incidence of colorectal cancer. Besides, the economic approach applied to convey health benefits by way of modifying the gut microbiota has been used to ferment dairy foods, which could markedly prevent colorectal cancer in the near future. It may thus be an effective strategy to integrate fermented dairy foods into eating habits for the early prevention of colorectal cancer. In parallel, we wished to see what role this meta-analysis could play in the dietary management of future outbreaks in colorectal neoplasms.

## Data Availability Statement

The raw data supporting the conclusions of this article will be made available by the authors, without undue reservation.

## Author Contributions

LL , JW, and XS conceived and designed the study. ZL and JH selected the studies and collected the data. RW, PL, and ZD analyzed data. All authors interpreted the results. ZL and JW drafted the paper. All authors revised the draft paper. All authors contributed to the article and approved the submitted version.

## Conflict of Interest

The authors declare that the research was conducted in the absence of any commercial or financial relationships that could be construed as a potential conflict of interest.

## Publisher’s Note

All claims expressed in this article are solely those of the authors and do not necessarily represent those of their affiliated organizations, or those of the publisher, the editors and the reviewers. Any product that may be evaluated in this article, or claim that may be made by its manufacturer, is not guaranteed or endorsed by the publisher.

## References

[1] Hyuna S Jacques F Rebecca LS Mathieu L Isabelle S Ahmedin J . Global Cancer Statistics 2020: GLOBOCAN Estimates of Incidence and Mortality Worldwide for 36 Cancers in 185 Countries. CA: A Cancer J Clin (2021) 71:209–49. doi: 10.3322/caac.21660 33538338

[2] Mingyang S Kana W Meyerhardt JA Shuji O Molin W Fuchs CS . Fiber Intake and Survival After Colorectal Cancer Diagnosis. JAMA Oncol (2018) 4:71–9. doi: 10.1001/jamaoncol.2017.3684 PMC5776713 29098294

[3] Cécile V Laurie J-C Manon L Emmanuel C Lemlih O Jocelyne D . Milk Polar Lipids Reduce Lipid Cardiovascular Risk Factors in Overweight Postmenopausal Women: Towards a Gut Sphingomyelin-Cholesterol Interplay. Gut (2020) 69:487–501. doi: 10.1136/gutjnl-2018-318155.4 31189655 PMC7034342

[4] Aryana KJ Olson DW . A 100-Year Review: Yogurt and Other Cultured Dairy Products. J Dairy Sci (2017) 100:9987–10013. doi: 10.3168/jds.2017-12981 29153184

[5] Winnie F Qing L Jun Y . Gut Microbiota Modulation: A Novel Strategy for Prevention and Treatment of Colorectal Cancer. Oncogene (2020) 39:4925-43. doi: 10.1038/s41388-020-1341-1 PMC7314664 32514151

[6] Cao Y Wu K Mehta R Drew DA Song M Lochhead P . Long-Term Use of Antibiotics and Risk of Colorectal Adenoma. Gut (2018) 67:672–8. doi: 10.1136/gutjnl-2016-313413 PMC5628103 28377387

[7] Zhenjiang X Rob K . Dietary Effects on Human Gut Microbiome Diversity. Br J Nutr (2015) 113 Suppl:1–5. doi: 10.1017/S0007114514004127 25498959 PMC4405705

[8] Laura B Nancy B Nerea B-T Núria R-E Jordi S-S . Association Between Dairy Product Consumption and Colorectal Cancer Risk in Adults: A Systematic Review and Meta-Analysis of Epidemiologic Studies. Adv Nutr (2019) 10:190–211. doi: 10.1093/advances/nmaa071 PMC6518136 31089733

[9] Ralston RA Truby H Palermo C Walker KZ . Colorectal Cancer and Nonfermented Milk, Solid Cheese, and Fermented Milk Consumption: A Systematic Review and Meta-Analysis of Prospective Studies. Crit Rev Food Sci Nutr (2014) 54:1167–79. doi: 10.1080/10408398.2011.629353 24499149

[10] Mazidi M Mikhailidis DP Sattar N Howard G Graham I Banach M . Consumption of Dairy Product and its Association With Total and Cause Specific Mortality - A Population-Based Cohort Study and Meta-Analysis. Clin Nutr (Edinburgh Scotland) (2019) 38:2833–45. doi: 10.1016/j.clnu.2018.12.015 30595374

[11] Kearney J Giovannucci E Rimm EB Ascherio A Stampfer MJ Colditz GA . Calcium, Vitamin D, and Dairy Foods and the Occurrence of Colon Cancer in Men. Am J Epidemiol (1996) 143:907–17. doi: 10.1093/oxfordjournals.aje.a008834 8610704

[12] Singh PN Gary FE . Dietary Risk Factors for Colon Cancer in a Low-Risk Population. Am J Epidemiol (1998) 148:761–74. doi: 10.1093/oxfordjournals.aje.a009697 9786231

[13] Terry P Baron JA Bergkvist L Holmberg L Wolk A . Dietary Calcium and Vitamin D Intake and Risk of Colorectal Cancer: A Prospective Cohort Study in Women. Nutr Cancer (2002) 43:39–46. doi: 10.1207/S15327914NC431_4 12467133

[14] Larcsson SC Bergkvist L Wolk A . High-Fat Dairy Food and Conjugated Linoleic Acid Intakes in Relation to Colorectal Cancer Incidence in the Swedish Mammography Cohort. Am J Clin Nutr (2005) 82:894–900. doi: 10.1093/ajcn/82.4.894 16210722

[15] Larcsson SC Leif B Jörgen R Edward G Alicja W . Calcium and Dairy Food Intakes are Inversely Associated With Colorectal Cancer Risk in the Cohort of Swedish Men. Narnia (2006) 83:667–73. doi: 10.1093/ajcn.83.3.667 16522915

[16] Valeria P Sabina S Franco B Paolo V Carlotta S Domenico P . Yogurt Consumption and Risk of Colorectal Cancer in the Italian European Prospective Investigation Into Cancer and Nutrition Cohort. Int J Cancer (2011) 129:2712–9. doi: 10.1002/ijc.26193 21607947

[17] Neil M Teresa N Pietro F Mazda J Bas B Guri S . Consumption of Dairy Products and Colorectal Cancer in the European Prospective Investigation Into Cancer and Nutrition (EPIC). PloS One (2013) 8:e72715–5. doi: 10.1371/journal.pone.0072715 PMC3759377 24023767

[18] Laura B Nancy B Guillermo M-S Estefania T Ramírez-Sabio JB Ramón E . Dairy Product Consumption and Risk of Colorectal Cancer in an Older Mediterranean Population at High Cardiovascular Risk. Int J Cancer (2018) 143:1356–66. doi: 10.1002/ijc.31540 29663376

[19] Matsumoto M Ishikawa S Nakamura Y Kayaba K Kajii E . Consumption of Dairy Products and Cancer Risks. Japan Epidemiol Assoc (2007) 17:38–44. doi: 10.2188/jea.17.38 PMC7058459 17420611

[20] Dik VK Murphy N Siersema PD Fedirko V Jenab M Kong SY . Prediagnostic Intake of Dairy Products and Dietary Calcium and Colorectal Cancer Survival–Results From the EPIC Cohort Study. Cancer Epidemiol Biomarkers Prev (2014) 23:1813–23. doi: 10.1158/1055-9965 24917183

[21] Kampman E van ‘t Veer P Hiddink GJ van Aken-Schneijder P Kok FJ Hermus RJ . Fermented Dairy Products, Dietary Calcium and Colon Cancer: A Case-Control Study in The Netherlands. Int J Cancer (1994) 59:170–6. doi: 10.1002/ijc.2910590205 7927914

[22] Kinany KE Deoula MMS Hatime Z Boudouaya HA Huybrechts I Asri AE . Consumption of Modern and Traditional Moroccan Dairy Products and Colorectal Cancer Risk: A Large Case Control Study. Eur J Nutr (2020) 59:953–63. doi: 10.1007/s00394-019-01954-1 30929068

[23] Shannon J White E Shattuck AL Potter JD . Relationship of Food Groups and Water Intake to Colon Cancer Risk. Cancer Epidemiol Biomarkers Prev (1996) 5:495–502. doi: 10.1016/S0003-4878(97)00007-0 8827352

[24] Boutron MC Faivre J Marteau P Couillault C Senesse P Quipourt V . Calcium, Phosphorus, Vitamin D, Dairy Products and Colorectal Carcinogenesis: A French Case–Control Study. Br J Cancer (1996) 74:145–51. doi: 10.1038/bjc.1996.330 PMC2074613 8679449

[25] Kampman E Slattery ML Caan B Potter JD . Calcium, Vitamin D, Sunshine Exposure, Dairy Products and Colon Cancer Risk (United States). Cancer Causes Control (2000) 11:459–66. doi: 10.1023/a:1008914108739 10877339

[26] Williams DC Satia JA Adair LS Steven J Galanko J Keku TO . Dietary Patterns, Food Groups, and Rectal Cancer Risk in Whites and African-Americans. Cancer Epidemiol Biomarkers Prev Publ Am Assoc Cancer Res Cosponsored by Am Soc Prev Oncol (2009) 18:1552–61. doi: 10.1158/1055-9965.EPI-08-1146 PMC2774490 19423533

[27] Zhuoyu S Peizhong PW Barbara R Michelle C Roger G Sharon B . Calcium and Vitamin D and Risk of Colorectal Cancer: Results From a Large Population-Based Case-Control Study in Newfoundland and Labrador and Ontario. Can J Public Health (2011) 102:382–9. doi: 10.1007/BF03404181 PMC6973686 22032106

[28] Plessas S Bosnea L Alexopoulos A Bezirtzoglou E . Potential Effects of Probiotics in Cheese and Yogurt Production: A Review. Eng Life Sci (2012) 12:433–40. doi: 10.1002/elsc.201100122

[29] Konishi H Fujiya M Tanaka H Ueno N Moriichi K Sasajima J . Probiotic-Derived Ferrichrome Inhibits Colon Cancer Progression *via* JNK-Mediated Apoptosis. Nat Commun (2016) 7:91–6. doi: 10.1038/ncomms12365 PMC4987524 27507542

[30] Qibiao W LaiHan LE . Association of Dietary Fiber and Yogurt Consumption With Lung Cancer Risk. JAMA Oncol (2020) 6:91–6. doi: 10.1001/jamaoncol.2020.0261 32191265

[31] Hassan EM Mansoor S Ammar HK Marjan M Samaneh M Fatemeh M . Calcium and Dairy Products in the Chemoprevention of Colorectal Adenomas: A Systematic Review and Meta-Analysis. Crit Rev Food Sci Nutr (2021), 21–5. doi: 10.1080/10408398.2021.1911927 33951958

[32] Lamprecht SA Lipkin M . Cellular Mechanisms of Calcium and Vitamin D in the Inhibition of Colorectal Carcinogenesis. Ann New York Acad Sci (2001) 952:73–87. doi: 10.1111/j.1749-6632.2001.tb02729.x 11795445

[33] Zhou X Cao L Jiang C Xie Y Cheng X Krausz KW . Pparα-UGT Axis Activation Represses Intestinal FXR-FGF15 Feedback Signalling and Exacerbates Experimental Colitis. Nat Commun (2014) 5:4573. doi: 10.1038/ncomms5573 25183423 PMC4164778

[34] Young HI Elvin K Adison W March JC Bentley WE Seng LY . Engineered Probiotic Escherichia Coli can Eliminate and Prevent Pseudomonas Aeruginosa Gut Infection in Animal Models. Nat Commun (2017) 8:15028. doi: 10.1038/ncomms15028 28398304 PMC5394271

[35] González S Fernández-Navarro T Arboleya S de los Reyes-Gavilán CG Salazar N Gueimonde M . Fermented Dairy Foods: Impact on Intestinal Microbiota and Health-Linked Biomarkers. Front Microbiol (2019) 10:1046. doi: 10.3389/fmicb.2019.01046 31191465 PMC6545342

[36] Louis P Hold GL Flint HJ . The Gut Microbiota, Bacterial Metabolites and Colorectal Cancer. Nat Rev Microbiol (2014) 12:661–72. doi: 10.1038/nrmicro3344 25198138

[37] Tianyu L Xueli S Samiullah K Yun L Zixuan G Chuqiao L . The Gut Microbiota at the Intersection of Bile Acids and Intestinal Carcinogenesis: An Old Story, Yet Mesmerizing. Int J Cancer (2020) 146:1780–90. doi: 10.1002/ijc.32563 31291465

[38] Quigley EMM . Prebiotics and Probiotics in Digestive Health. Clin Gastroenterol Hepatol (2019) 17:333–44. doi: 10.1016/j.cgh.2018.09.028 30267869

[39] Gori S Inno A Belluomini L Bocus P Bisoffi Z Russo A . Gut Microbiota and Cancer: How Gut Microbiota Modulates Activity, Efficacy and Toxicity of Antitumoral Therapy. Crit Rev Oncol / Hematol (2019) 143:139–47. doi: 10.1016/j.critrevonc.2019.09.003 31634731

[40] Ashutosh T Jayalaxmi D Sudhakar K Prachetha K Deviyani M Shantibhusan S . Probiotics: A Promising Candidate for Management of Colorectal Cancer. Cancers (2021) 13:3178–8. doi: 10.3390/cancers13133178 PMC8268640 34202265

